# Association of Long-term Exposure to Particulate Air Pollution With Cardiovascular Events in California

**DOI:** 10.1001/jamanetworkopen.2023.0561

**Published:** 2023-02-24

**Authors:** Stacey E. Alexeeff, Kamala Deosaransingh, Stephen Van Den Eeden, Joel Schwartz, Noelle S. Liao, Stephen Sidney

**Affiliations:** 1 Kaiser Permanente Division of Research, Oakland, California; 2 Harvard T.H. Chan School of Public Health, Boston, Massachusetts

## Abstract

**Importance:**

Long-term exposure to fine particulate air pollution (PM_2.5_) is a known risk factor for cardiovascular events, but controversy remains as to whether the current National Ambient Air Quality Standard (12 μg/m^3^ for 1-year mean PM_2.5_) is sufficiently protective.

**Objective:**

To evaluate the associations between long-term fine particulate air pollution and cardiovascular events using electronic health record and geocoded address data.

**Design, Setting, and Participants:**

This retrospective cohort study included adults in the Kaiser Permanente Northern California integrated health care system during 2007 to 2016 and followed for up to 10 years. Study participants had no prior stroke or acute myocardial infarction (AMI), and lived in Northern California for at least 1 year. Analyses were conducted January 2020 to December 2022.

**Exposure:**

Long-term exposure to PM_2.5_. Individual-level time-varying 1-year mean PM_2.5_ exposures for every study participant were updated monthly from baseline through the end of follow-up, accounting for address changes.

**Main Outcomes and Measures:**

Incident AMI, ischemic heart disease (IHD) mortality, and cardiovascular disease (CVD) mortality. Cox proportional hazards models were fit with age as time scale, adjusted for sex, race and ethnicity, socioeconomic status, smoking, body mass index, baseline comorbidities, and baseline medication use. Associations below the current regulation limit were also examined.

**Results:**

The study cohort included 3.7 million adults (mean [SD] age: 41.1 [17.2] years; 1 992 058 [52.5%] female, 20 205 [0.5%] American Indian or Alaskan Native, 714 043 [18.8%] Asian, 287 980 [7.6%] Black, 696 796 [18.4%] Hispanic, 174 261 [4.6%] multiracial, 1 904 793 [50.2%] White). There was a 12% (95% CI, 7%-18%) increased risk of incident AMI, a 21% (95% CI, 13%-30%) increased risk of IHD mortality, and an 8% (95% CI, 3%-13%) increased risk of CVD mortality associated with a 10 μg/m^3^ increase in 1-year mean PM_2.5_. PM_2.5_ exposure at moderate concentrations (10.0 to 11.9 μg/m^3^) was associated with increased risks of incident AMI (6% [95% CI, 3%-10%]) and IHD mortality (7% [95% CI, 2%-12%]) compared with low concentrations (less than 8 μg/m^3^).

**Conclusions and Relevance:**

In this study, long-term PM_2.5_ exposure at moderate concentrations was associated with increased risks of incident AMI, IHD mortality, and CVD mortality. This study’s findings add to the evidence that the current regulatory standard is not sufficiently protective.

## Key Points

**Question:**

What are the long-term cardiovascular health associations of fine particulate air pollution (PM_2.5_)?

**Findings:**

In a diverse cohort of 3.7 million adults, this cohort study found that long-term PM_2.5_ exposure was associated with an increased risk of incident acute myocardial infarction, ischemic heart disease mortality, and cardiovascular disease mortality, and these associations were more pronounced in low socioeconomic status communities. This study also found evidence of associations at moderate concentrations of PM_2.5_ below the current regulatory standard of 12 μg/m^3^.

**Meaning:**

This study’s results add to the growing evidence that long-term PM_2.5_ exposure is associated with increased risk of cardiovascular events and that the current regulatory standard of 12 μg/m^3^ is not sufficiently protective.

## Teaser

This cohort study examines associations between long-term fine particulate air pollution and cardiovascular events using electronic health record and geocoded address data.

## Introduction

Fine particulate air pollution (PM_2.5_) is recognized as a risk factor for cardiovascular events and mortality.^1,2,3^ Long-term PM_2.5_ exposures (1 year or more) have shown strong associations with cardiovascular mortality, including ischemic heart disease (IHD) mortality.^4,5^ However, several key gaps in knowledge remain. First, a recent meta-analysis found much weaker evidence for the association of long-term PM_2.5_ with incident AMI than with cardiovascular mortality outcomes.^4^ Incident AMI is more difficult to study than mortality since medical record information is needed to determine the AMI, and past medical history is needed to discern incidence. Relatively few studies in the US have examined the association of long-term PM_2.5_ with incident AMI.^4^ Second, there is a gap in knowledge on susceptibility factors such as age, sex, race and ethnicity, and socioeconomic status (SES), where previous studies examining these factors have reported mixed and inconsistent results.^6,7,8,9,10,11,12^ Finally, controversy still remains as to whether the current US National Ambient Air Quality Standard of 12 μg/m^3^ for annual mean exposures is sufficiently protective of adverse health effects.^13^

In the US, rigorous prospective cohort studies are the foundation of our knowledge of the cardiovascular health effects of long-term PM_2.5_.^14,15,16,17,18^ Recently, studies of Medicare claims data have leveraged the use of big data in air pollution epidemiology research.^19,20,21^ Although these studies offer increased power, there is a potential for confounding and bias due to the lack of data on important covariates (eg, smoking, comorbidities) and the use of aggregate zip codes to determine exposures rather than precise geocoded address locations.

We conducted a retrospective cohort study of 3.7 million adults followed up for up to 10 years to quantify the association of long-term fine particulate air pollution with incident AMI, IHD mortality, and CVD mortality. This study represents an important contribution to the field by extracting key information previously only available in smaller prospective cohort studies of air pollution: geocoded longitudinal residential address data and detailed health information on important confounders such as smoking status, body mass index (BMI, calculated as weight in kilograms divided by height in meters squared), and comorbidity diagnoses. To our knowledge, this is the first US study of long-term PM_2.5_ exposure and cardiovascular events that includes more than 1 million people with individual-level geocoded address data. We constructed time-updated 1-year mean PM_2.5_ exposures for every study participant. We also investigated whether associations varied by age, sex, race and ethnicity, and neighborhood SES, and whether these associations persisted at levels below the standard of 12 μg/m^3^.

## Methods

### Study Cohort

This retrospective cohort study during 2007 to 2016 included adults who were members of the Kaiser Permanente Northern California (KPNC) health plan. KPNC is a large, integrated health care system that provides comprehensive medical services to more than 4 million members through a nonprofit health plan and nonprofit hospitals and outpatient clinics. Participants were included who met the following criteria: an adult (aged 18 years or older), at least 1 year of KPNC health plan membership, at least 1 outpatient utilization, lived in the Northern California region for at least 1 year, and had a home address that was successfully geocoded and linked to the air pollution data. Study follow-up began on January 1, 2007, with participants entering the study on the first day that all inclusion criteria were met. Follow-up continued for up to 10 years to the first of the following dates: end of membership, relocation out of study region or to an address that could not be successfully geocoded, death, or end of study (December 31, 2016). This cohort was assembled for the Particulate Air Pollution, Cardiovascular Events, and Susceptibility Factors (PACES) study. The institutional review board at the Kaiser Foundation Research Institute approved this study and waived informed consent. Study procedures meet Health Insurance Portability and Accountability Act requirements and the 42 CFR Part 2 regarding medical records. On enrollment in the health plan, all KPNC members are informed that their data may be used for research. This study follows the Strengthening the Reporting of Observational Studies in Epidemiology (STROBE) reporting guideline.

### Air Pollution Exposures

We constructed individual-level time-updated 1-year mean PM_2.5_ exposures for every study participant, updated monthly from baseline through the end of follow-up, accounting for address changes. Thus, a participant followed for all 10 years would have 120 individual-level time-updated measurements of 1-year mean air pollution. We extracted residential address histories of all cohort participants from 1 year before the study entry date to the end of follow-up from the KPNC historical and current residential address databases. Each address was geocoded to the latitude, longitude coordinates using ArcGIS, and coordinates were linked with the PM_2.5_ exposure data. PM_2.5_ exposures were obtained from a validated ensemble model with outstanding cross-validated model performance (*R*^2^ of 0.89 for 1-year mean PM_2.5_ predictions).^22^ The ensemble model integrated 3 machine learning algorithms and combined discrete PM_2.5_ daily ground monitoring data with more than 100 predictor variables including satellite-based aerosol optical depth measurements, absorbing aerosol index data, satellite-based surface reflectance data, chemical transport model outputs, meteorologic data, and land-use data.^22^

### Cardiovascular Event Outcomes

We examined 3 primary cardiovascular event end points: incident AMI, IHD mortality, and CVD mortality. An incident AMI event was defined as an inpatient hospitalization with a principal discharge diagnosis of AMI based on *International Classification of Diseases, Ninth Revision (ICD-9)* and *International Statistical Classification of Diseases and Related Health Problems, Tenth Revision (ICD-10)* codes (*ICD-9*: 410.x; *ICD-10*: I21.x-I23.x), using previously validated methods.^23,24^ To ensure capture of incident events, participants with any history of AMI before study start were excluded. Secondary analyses examined AMI subtypes: ST-Segment Elevation Myocardial Infarction (STEMI) and non–ST-elevation myocardial infarction (NSTEMI). Cause of death data was obtained from official state of California death certificates and the National Death Index. IHD mortality was defined by cause of death codes (*ICD-9*: 410.x-414.x; *ICD-10*: I20.x-I25.x) and CVD mortality was defined by cause of death codes (*ICD-9*: 400.x-440.x; *ICD-10*: I10.x-I70.x), following previous studies.^25,26^

### Covariates

Data on age, sex, race and ethnicity, smoking, BMI, comorbidities, insurance type, and medication use were obtained from the KPNC electronic health record (EHR). Race and ethnicity were self-reported and recorded in the EHR. Race and ethnicity data were included in the study to account for differences in risk of cardiovascular events and to assess potential differences in susceptibility to PM_2.5_. BMI was categorized as underweight (less than 18.5), normal (18.5 to 24.9), overweight (25.0-29.9), or obese (30 or higher). Missing data were imputed for sex (less than 0.1%), race and ethnicity (8.6%), smoking (5.2%), and BMI (4.7%) using the fully conditional specification method (see eMethods in Supplement 1).^27^ Comorbidities (hypertension, hyperlipidemia, diabetes, chronic obstructive pulmonary disease [COPD]) were determined from the EHR. Diabetes was determined from the previously validated KPNC Diabetes registry, based on diagnoses, medications, and laboratory results documented in the EHR. Hypertension was determined from diagnosis codes (*ICD-9*: 401.x-404.x; *ICD-10*: I10.x-I15.x). Hypertensive and statin medication use at baseline were defined as having at least 1 prescription fill in the last 120 days based on pharmacy data. Neighborhood high school education was obtained from Census American Community Survey data at the block group level and used as a measure of neighborhood SES. Medicaid insurance was used as an indicator of low SES at the individual level.

### Statistical Analysis

We fit Cox proportional hazards models and estimated the hazard ratio (HR) and corresponding 95% CI to quantify the association between long-term PM_2.5_ exposure and each cardiovascular outcome. We used age as the time scale to flexibly control for age and modeled PM_2.5_ exposure as a time-updated variable (eFigure 1 in Supplement 1). We assessed departures from the proportional hazards assumption by including an interaction term with age centered at 65 years.^28^ We fit a set of nested models with increasing levels of adjustment, with covariates chosen a priori based on previous epidemiologic studies of air pollution and CVD events^17,29,30^ or based on well-established associations with CVD.^27^ Model 1 (minimally adjusted model) adjusted for age, sex, and race and ethnicity. Model 2 adjusted for age, sex, race and ethnicity, and SES. Model 3 (fully adjusted model) adjusted for age, sex, race and ethnicity, SES, smoking, BMI, baseline comorbidities, and baseline medication use. All models accounted for the competing risk of death by estimating the cause-specific hazard.^31^ For incident AMI, death from any cause was considered a competing risk; for IHD and CVD mortality, death from any other cause was considered a competing risk. We conducted several sensitivity analyses. Each covariate was sequentially added in model 3 to see the degree of confounding. We adjusted for calendar year of cohort entry to compare with prior studies, but not in main models because it can induce bias in association estimates in air pollution studies.^32^ To investigate the potential association of informative censoring, we fit models with inverse probability of censoring weighting (IPCW).^33^ We tested for nonlinearity in the association between PM_2.5_ and risk of each outcome by including a quadratic term. We assessed effect modification by age (less than 65 years vs at least 65 years), sex, race and ethnicity, neighborhood education, and smoking using interaction terms. We examined effects of PM_2.5_ exposure categorically at levels below the current regulation limit: low (less than 8.0 μg/m^3^), low-moderate (8.0 to 9.9 μg/m^3^), moderate (10.0 to 11.9 μg/m^3^), high (12.0 to 13.9 μg/m^3^), and very high (at least 14.0 μg/m^3^). Cutoffs were chosen based on the current annual US and state regulation limit of 12.0 μg/m^3,34,35^ and based on the annual limit of 10.0 μg/m^3^ recommended by WHO since 2006 and recently proposed as the new maximum concentration in the UK.^36,37^ We then used equally spaced units of 2.0 μg/m^3^ for both biologic and policy relevance to PM_2.5_ measures, with 8.0 μg/m^3^ chosen as the lowest exposure cutoff supported by the distribution of our data (470 716 participants [12.4%] had PM_2.5_ exposures less than 8.0 μg/m^3^ at baseline). A level of α = .05 was used to determine statistical significance. Analyses were conducted by S.A. and K.D. from January 2020 to December 2022 using H_2_O version 3.32.0.4 (H20.ai), R version 3.6.0 (R Project for Statistical Computing), and SAS version 9.4 (SAS Institute).

## Results

**Figure 1.**
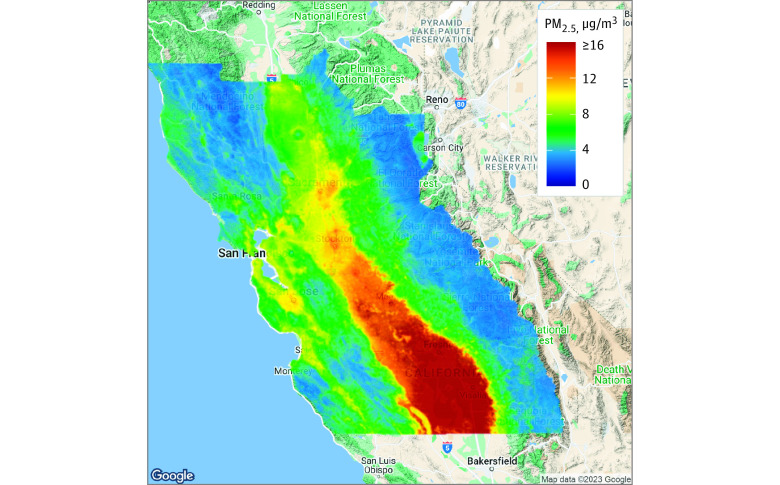
Distribution of 1-Year Mean PM_2.5_ Across the 35-County Study Region in Northern California for 2007

This retrospective cohort study included 3 798 078 adults followed up for up to 10 years. Participants had a mean (SD) age of 41.1 (17.2) years; 1 992 058 [52.5%] were female; and the cohort was diverse with 20 205 [0.5%] American Indian or Alaskan Native, 714 043 [18.8%] Asian, 287 980 [7.6%] Black, 696 796 [18.4%] Hispanic, 174 261 [4.6%] multiracial, and1 904 793 [50.2%] White (Table 1). The final analytic data set included 231 million rows, one for each person-month of follow-up. Figure 1 shows the substantial variation in 1-year mean PM_2.5_ distribution across the study region.

**Table 1.** Characteristics of the PACES Cohort at Baseline

Characteristic	PACES Cohort, No. (%) (N = 3 798 078)
Sex	
Female	1 992 058 (52.4)
Male	1 806 020 (47.6)
Age, mean (SD), y	41.1 (17.2)
Race and ethnicity	
American Indian or Alaska Native	20 205 (0.53)
Asian	714 043 (18.8)
Black	287 980 (7.6)
Hispanic, White	696 796 (18.4)
Multiracial	174 261 (4.6)
White, non-Hispanic	1 904 793 (50.2)
Neighborhood education (less than high school education)	
<10%	1 838 172 (48.40)
10 to <20%	1 001 613 (25.2)
≥20%	958 293 (25.2)
Smoking status	
Never smoked	2 497 571 (65.8)
Former smoker	611 156 (16.1)
Current	689 351 (18.2)
BMI	
Underweight (<18.5)	80 722 (2.1)
Normal (18.5-24.9)	1 321 150 (34.8)
Overweight (25.0-29.9)	1 269 552 (33.4)
Obese (≥30.0)	1 126 654 (29.7)
Comorbidities	
Hypertension	640 293 (16.9)
Hyperlipidemia	666 747 (17.6)
Diabetes	227 264 (6.0)
COPD	146 853 (3.9)
Medications	
Hypertension medication^a^	668 453 (93.1)
Statin medication	468 629 (12.3)
1-y mean PM_2.5 _at baseline	
<8.0 μg/m^3^	470 716 (12.4)
8.0-9.9 μg/m^3^	1 333 593 (35.1)
10.0-11.9 μg/m^3^	1 405 322 (37.0)
12.0-13.9 μg/m^3^	37 3931 (9.9)
≥14.0 μg/m^3^	214 516 (5.7)

Abbreviations: BMI, body mass index (calculated as weight in kilograms divided by height in meters squared); COPD, chronic obstructive pulmonary disease; PACES, the Particulate Air Pollution, Cardiovascular Events, and Susceptibility Factors study; PM_2.5_, particulate matter less than 2.5 microns in diameter.

^a^ Among those with hypertension only.

We found that long-term PM_2.5_ exposure was associated with increased risk of each outcome in all models (Table 2). In model 1 (minimally adjusted), we found: a 38% (95% CI, 32%-45%) increased risk of incident AMI, a 50% (95% CI, 40%-60%) increased risk of IHD mortality, and a 30% (95% CI, 24%-36%) increased risk of CVD mortality per 10 μg/m^3^ increase in 1-year mean PM_2.5_. When sequentially adding covariates, we observed confounding by SES and baseline comorbidities, and negligible confounding by smoking, BMI, and medication use (models 2.1 to 2.3; eTable 1 in Supplement 1). In model 3 (fully adjusted), we found a 12% (95% CI, 7%-18%) increased risk of incident AMI, a 21% (95% CI, 13%-30%) increased risk of IHD mortality, and an 8% (95% CI, 3%-13%) increased risk of CVD mortality per 10 μg/m^3^ increase in 1-year mean PM_2.5_. eFigures 2, 3, and 4 in Supplement 1 show a comparison of these results to the individual and combined estimates of previous studies in a recently published meta-analysis, demonstrating that our results were very consistent, with excellent precision due to our large cohort.

**Table 2.** Relative Risk of Cardiovascular Mortality, IHD, and Incident AMI per 10 μg/m^3^ Increase in 1-Year Mean PM_2.5_ Exposure Among 3.7 Million Participants in the Particulate Air Pollution, Cardiovascular Events, and Susceptibility Factors Cohort^a^

Model	Covariates	HR (95% CI)		
		Incident AMI (30 165 events)	IHD Mortality (14 278 events)	CVD Mortality (31 284 events)
Model 1	Age, sex, race and ethnicity	1.38 (1.32-1.45)	1.50 (1.40-1.60)	1.30 (1.24-1.36)
Model 2	Age, sex, race and ethnicity, SES	1.27 (1.21-1.33)	1.34 (1.25-1.43)	1.18 (1.13-1.24)
Model 3	Age, sex, race and ethnicity, SES, smoking, BMI, baseline comorbidities, baseline medication use	1.12 (1.07-1.18)	1.21 (1.13-1.30)	1.08 (1.03-1.13)

Abbreviations: AMI, acute myocardial infarction; BMI, body mass index; CVD, cardiovascular disease; IHD, ischemic heart disease; SES, socioeconomic status.

^a^ Data presented as HR (95%). All models adjusted for age by using age as the time scale in the Cox Proportional Hazards models.

Results were very similar in sensitivity analyses adjusting for calendar year and when using IPCW (eTable 1 in Supplement 1). We found that follow-up time differed by age and SES (eTable 2 in Supplement 1). The proportional hazards assumption was satisfied for AMI and for IHD mortality (AMI *P* for interaction = .37; IHD *P* for interaction = .24), but we identified a departure from the proportional hazards assumption for CVD mortality (*P* for interaction < .001). When hazards are nonproportional, the overall HR can be interpreted as a weighted mean of the time-varying hazard ratios, averaged over the event times.^28,38^ Because age was the time scale, the overall HR for CVD mortality is interpreted as a weighted mean of the time-varying hazard ratios, averaged over the ages at CVD death. The interaction with continuous age found that the HR varied from 1.35 at age 50 years, 1.25 at age 60 years, 1.16 at age 70 years, and 1.08 at age 80 years. The mean (SD) age at CVD death in our study was 79.6 (13.1) years of age, so we see that the estimated HR at age 80 also reflects the overall HR reported in Table 2. All models accounted for the competing risk of death and estimated the cause-specific hazard; for these competing risks, we found a 5% (95% CI, 2%-7%) increased risk of death from any cause and a 4% (95% CI, 1%-7%) increased risk of death from any non-CVD cause per 10 μg/m^3^ increase in 1-year mean PM_2.5_ in the fully adjusted model.

**Figure 2.**
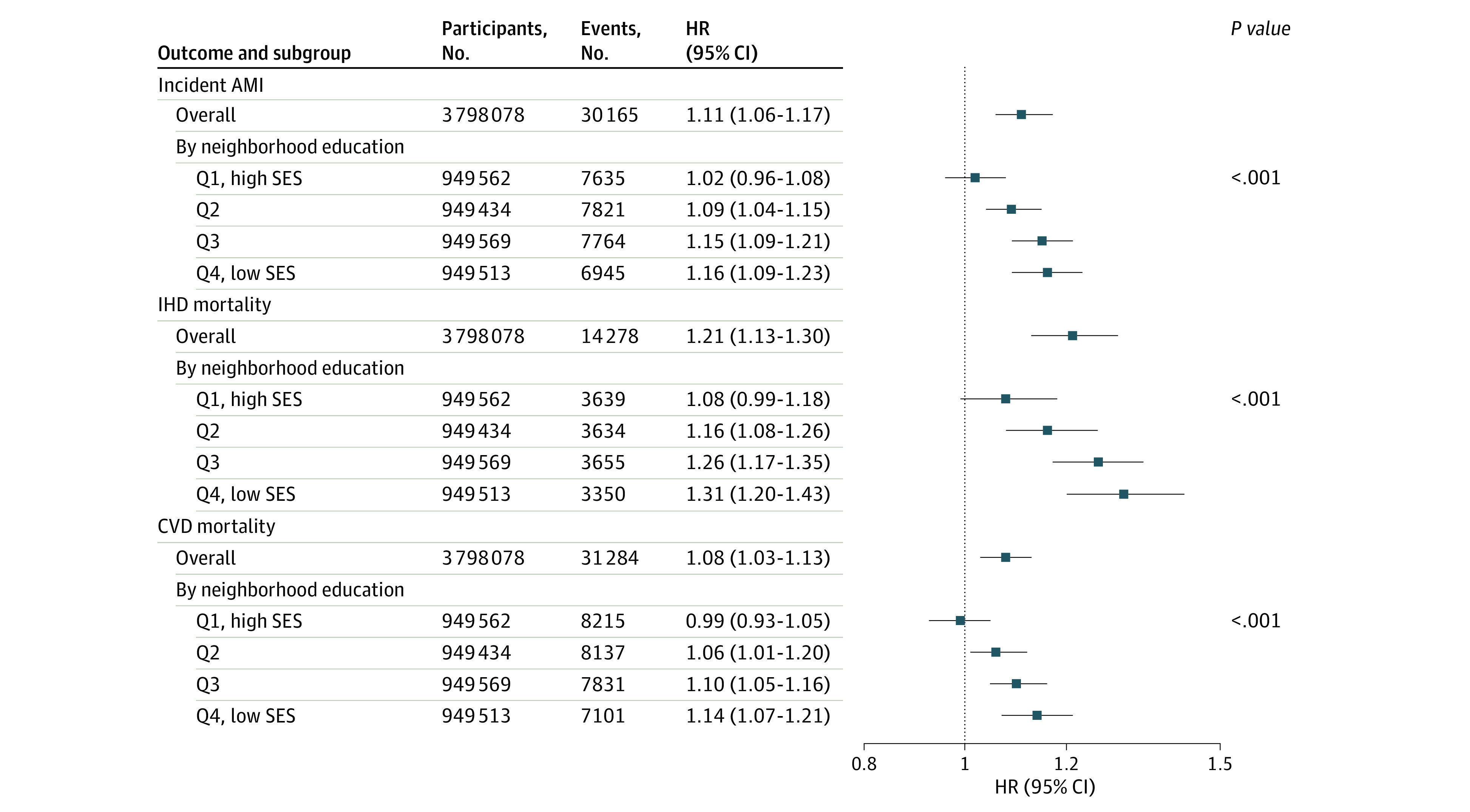
Relative Risk of Incident Acute Myocardial Infarction (AMI), Ischemic Heart Disease (IHD) Mortality, and Cardiovascular Disease (CVD) Mortality Associated With 1-Year Mean Particulate Air Pollution Exposure, Overall And by Neighborhood Education Level, Among 3.7 Million Participants in the Particulate Air Pollution, Cardiovascular Events, and Susceptibility Factors Cohort Adjusted for age, sex, race and ethnicity, socioeconomic status, smoking, body mass index, baseline comorbidities, baseline medication use.* P* values are for interaction with neighborhood education. SES indicates socioeconomic status, which was measured by neighborhood education.

We found evidence of effect modification by neighborhood SES for all outcomes, with larger PM_2.5_ associations among those living in low SES neighborhoods compared with high SES neighborhoods (Figure 2; eTable 3 in Supplement 1). In sensitivity analyses using neighborhood income, we found similar effect modification results for IHD and CVD mortality, but no differences by neighborhood income for incident AMI (eTable 4 in Supplement 1). There was no evidence of increased risk for Black or Hispanic participants compared with White participants, and no effect modification by smoking status (eTable 3 in Supplement 1). Relative risks were larger at ages less than 65 years compared with ages greater than 65 years for CVD mortality but not for IHD mortality or incident AMI (eTable 3 in Supplement 1). Relative risks were larger among female participants compared with male participants for IHD mortality but not for CVD mortality or incident AMI (eTable 3 in Supplement 1). Secondary analyses of AMI subtypes found associations for NSTEMI AMIs (HR, 1.16 [95% CI, 1.09-1.23]) but not for STEMI AMIs (HR, 1.04 [95%CI: 0.95-1.14]) per 10 μg/m^3^ increase in 1-year mean PM_2.5_.

**Figure 3.**
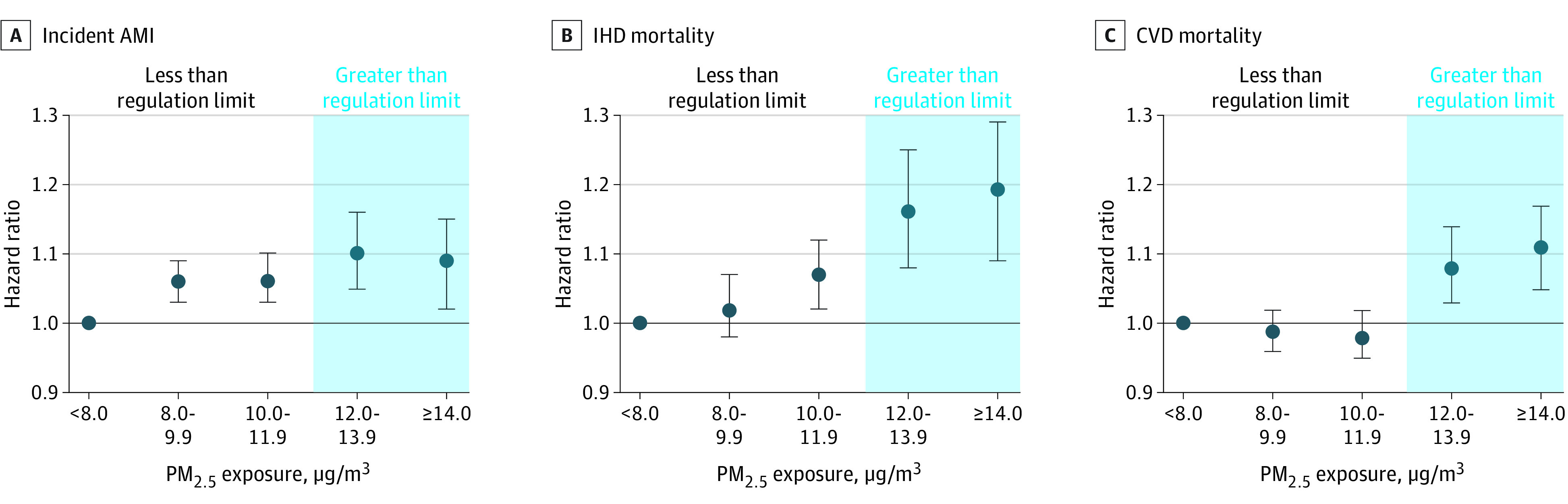
Relative Risk of Cardiovascular Disease (CVD) Mortality, Ischemic Heart Disease (IHD) Mortality, and Incident Acute Myocardial Infarction (AMI) Associated With 1-Year Mean PM_2.5_ Exposure, in Categories Above and Below the Regulation Limit, Among 3.7 Million Participants in the Particulate Air Pollution, Cardiovascular Events, and Susceptibility Factors Cohort PM_2.5_ indicates particulate air pollution less than 2.5 microns in diameter.

When examining associations in categories below the current regulation limit, PM_2.5_ exposure at moderate concentrations (10.0 to 11.9 μg/m^3^) compared with low concentrations (less than 8.0 μg/m^3^) was associated with a 6% (95% CI, 3%-10%) increased risk of incident AMI and a 7% (95% CI, 2%-12%) risk of IHD mortality, but no increase in the risk of CVD mortality (Figure 3; eTable 5 in Supplement 1). In sensitivity analyses examining incident AMI associations within the range of 8.0 to 9.9 μg/m^3^ compared with low concentrations (<8.0 μg/m^3^), we found that PM_2.5_ exposure at concentrations of 8.0 to 8.9 μg/m^3^ was associated with a 5% (95% CI, 2%-9%) increased risk of incident AMI and that PM_2.5_ exposure at concentrations of 9.0 to 9.9 μg/m^3^ was associated with a 6% (95% CI, 3%-10%) increased risk of incident AMI.

Above the current regulation limit, PM_2.5_ exposure at high concentrations (12.0 to 13.9 μg/m^3^) was associated with a 10% (95% CI, 5%-16%) increased risk of incident AMI (95% CI, 5%-16%), a 16% (95% CI, 8%-25%) increased risk of IHD mortality, and an 8% (95% CI, 3%-14%) increased risk of CVD mortality, compared with low concentrations (less than 8.0 μg/m^3^). PM_2.5_ had a linear association with IHD mortality, and nonlinear associations with incident AMI and CVD mortality (Figure 3; eTable 6 in Supplement 1).

## Discussion

In this retrospective cohort study of 3.7 million adults using electronic health records, we found that long-term PM_2.5_ exposure was associated with an increased risk of incident AMI, IHD mortality, and CVD mortality. Low SES communities had a higher risk. Associations with incident AMI and IHD mortality were present at moderate concentrations of PM_2.5_ below the current regulatory standard. By using EHR data, this study combines the large sample size with the advantages of smaller prospective cohorts: the ability to use residential address-based air pollution exposures and to control for medication, baseline comorbidities, smoking, and BMI.

Our study adds important evidence to the literature on long-term PM_2.5_ and incident AMI, which has been inconsistent. In a recent meta-analysis, only 4 of 11 studies on long-term PM_2.5_ and incident AMI reported an increased risk, whereas 6 studies reported no statistically significant association, and 1 study reported a negative association.^4^ The combined relative risk of AMI was highly suggestive of an association but did not reach statistical significance (1.08 [95% CI, 0.99-1.18] per 10 μg/m^3^ increase in long-term PM_2.5_).^4^ Our finding of an increased risk of incident AMI of 1.12 (95% CI: 1.07, 1.18) per 10 μg/m^3^ increase in long-term PM_2.5_ is very similar in magnitude, with a much narrower CI.

Our findings of increased risk of IHD mortality and CVD mortality are very consistent with previous literature from numerous cohort studies, demonstrating the success of our novel big data approach linking detailed EHR data and individual-level PM_2.5_ data. Our relative risk of IHD mortality (1.21 [95% CI, 1.13-1.30] per 10 μg/m^3^ ) was extremely similar to the combined association reported in a recent meta-analysis (1.23 [95% CI, 1.15-1.32] per 10 μg/m^3^).^4^ Similarly, our relative risk of CVD mortality (1.08 [95% CI, 1.03-1.13] per 10 μg/m^3^) was also consistent with the combined relative risk of CVD mortality reported in 2 meta-analyses (1.11 [95% CI, 1.05-1.16] and 1.14 [95% CI, 1.08-1.21] and per 10 μg/m^3^ increase in long-term PM_2.5_), noting that those meta-analyses found high-heterogeneity in results across studies (*I*^2^ = 98.6% and *I*^2^ = 61.2%).^4,5^

At moderate concentrations of long-term PM_2.5_ exposure, we also found evidence of an increased risk of incident AMI and IHD mortality, but not CVD mortality. Our findings add to growing evidence that the current regulatory standard of 12 μg/m^3^ is not sufficiently protective of human health.^3,13^ Notably, most of the evidence of associations below 12 μg/m^3^ is based on mortality studies.^19,39^ To our knowledge, this is the first study to show an increased risk of incident AMI at moderate concentrations of long-term PM_2.5_ exposure.

We found strong evidence of effect modification by neighborhood SES across all 3 CVD outcomes, with greater susceptibility among those living in low SES neighborhoods compared with high SES neighborhoods. This is an important contribution because most previous studies of long-term PM_2.5_ exposure have only reported effect modification by neighborhood SES for associations with all-cause mortality and combined end points of any CVD event,^40,41,42^ with only 1 study previously reporting stronger associations in lower income neighborhoods for incident AMI.^11^ Our study shows the consistency of this effect modification by neighborhood education across 3 CVD end points. We found inconsistent or null effect modification by age, sex, race and ethnicity, and smoking, similar to the mixed and inconclusive results reported in previous studies.^6,7,8,9,10,11,12^

### Strengths and Limitations

This study has several strengths and limitations. Major strengths are the use of a large, diverse, representative cohort with well-characterized health data available from EHRs, and the use of individual-level, time-updated PM_2.5_ exposures based on geocoded residential addresses. Our study focused on 1-year mean exposures because that is the long-term average regulated in both California and the United States. Using geocoded residential addresses reduces bias compared with using county-level or zip code–level averages.^43^ However, the lack of individual-level time-activity data and time outdoors can still induce a combination of Berkson and/or classical measurement error, which may bias associations in either direction.^44,45,46^ Other studies have found that adults in California typically spend at least 65% of daily time at their home residence,^47^ and that high air exchange rates can produce similar ambient contributions of PM_2.5_ indoors, outdoors, and personal concentrations.^48,49^ Our study controls for key confounders at baseline, including smoking, BMI, comorbidities, and medication use. Because diabetes, hypertension, hyperlipidemia, and COPD have been associated with PM_2.5_ exposure, they may be part of the causal pathway between exposure and outcome.^3^ Thus, we adjusted for comorbidities and medication use at baseline only, and our study focused on time-updated 1-year mean PM_2.5_ exposure. The total effect of multiyear, long-term PM_2.5_ exposures may be larger. We did not have data available on physical activity, diet, alcohol use, marital status, or detailed smoking history, which is a limitation. We imputed missing covariates using single imputation, which reduces bias compared with a complete case analysis; however, a limitation of single imputation is that the standard errors may be underestimated compared with using multiple imputation.

## Conclusions

In this retrospective cohort study of 3.7 million adults linking detailed EHR data, geocoded address data, and time-varying 1-year mean PM_2.5_ exposures, we found that long-term PM_2.5_ exposure was associated with increased risks of incident AMI, IHD mortality, and CVD mortality, and that neighborhood SES was an important effect modifier. We also found associations at moderate vs low concentrations of PM_2.5_, adding to the evidence that the current regulatory standard of 12 μg/m^3^ is not sufficiently protective of health.

## Notes

Supplement 1. **eFigure 1.** Survival Analysis Study Designs Illustrating Age as Time Scale, Time on Study, Baseline Air Pollution Exposure, and Time-Updated Air Pollution Exposure **eFigure 2.** PACES Estimated CVD Mortality Effect Compared to Previous Studies in a Recently Published Meta-analysis **eFigure 3.** PACES Estimated IHD Mortality Effect Compared to Previous Studies in a Recently Published Meta-analysis **eFigure 4.** PACES Estimated Incident AMI Effect Compared to Previous Studies in a Recently Published Meta-analysis **eTable 1.** Sensitivity Analyses Showing the Relative Risk of Each Outcome Associated With a 10 μg/m3 Increase in 1-Year Mean PM2.5 Exposure for Different Models **eTable 2.** Follow-up Time in Years, Overall and by Age and SES **eTable 3.** Relative Risk of Each Outcome Associated With 1-Year Mean PM2.5 Exposure, Overall and by Subgroups for Age, Sex, Race, Neighborhood Education, and Smoking Status **eTable 4.** Sensitivity Analysis of the Relative Risk of Incident AMI, IHD Mortality, Cardiovascular Mortality, and Associated With 1-Year Mean PM2.5 Exposure, in Quartiles of Neighborhood Median Income, Among 3.7 Million Subjects in the PACES Cohort **eTable 5.** Relative Risk of Cardiovascular Mortality, IHD Mortality, and Incident AMI Associated With 1-Year Mean PM2.5 Exposure, in Categories Above and Below the Regulation Limit, Among 3.7 Million Subjects in the PACES Cohort **eTable 6.** Assessment of Linearity of Associations by Adding a Quadratic Term for 1-Year Mean PM2.5 Exposure to the Linear Associations in the Fully Adjusted Models (Model 3), Among 3.7 Million Subjects in the PACES Cohort **eMethods.** Supplementary Methods

Supplement 2.
Data Sharing Statement
