# Supplementary material for: Association of Long-term Exposure to Particulate Air Pollution With Cardiovascular Events in California
Source: JAMA Netw Open. 2023 Feb 24;6(2):e230561. doi: 10.1001/jamanetworkopen.2023.0561 (PMC9958530; doi:10.1001/jamanetworkopen.2023.0561)
Supplement: Supplement 1. — eFigure 1. Survival Analysis Study Designs Illustrating Age as Time Scale, Time on Study, Baseline Air Pollution Exposure, and Time-Updated Air Pollution Exposure eFigure 2. PACES Estimated CVD Mortality Effect Compared to Previous Studies in a Recently Published Meta-analysis eFigure 3. PACES Estimated IHD Mortality Effect Compared to Previous Studies in a Recently Published Meta-analysis eFigure 4. PACES Estimated Incident AMI Effect Compared to Previous Studies in a Recently Published Meta-analysis eTable 1. Sensitivity Analyses Showing the Relative Risk of Each Outcome Associated With a 10 μg/m3 Increase in 1-Year Mean PM2.5 Exposure for Different Models eTable 2. Follow-up Time in Years, Overall and by Age and SES eTable 3. Relative Risk of Each Outcome Associated With 1-Year Mean PM2.5 Exposure, Overall and by Subgroups for Age, Sex, Race, Neighborhood Education, and Smoking Status eTable 4. Sensitivity Analysis of the Relative Risk of Incident AMI, IHD Mortality, Cardiovascular Mortality, and Associated With 1-Year Mean PM2.5 Exposure, in Quartiles of Neighborhood Median Income, Among 3.7 Million Subjects in the PACES Cohort eTable 5. Relative Risk of Cardiovascular Mortality, IHD Mortality, and Incident AMI Associated With 1-Year Mean PM2.5 Exposure, in Categories Above and Below the Regulation Limit, Among 3.7 Million Subjects in the PACES Cohort eTable 6. Assessment of Linearity of Associations by Adding a Quadratic Term for 1-Year Mean PM2.5 Exposure to the Linear Associations in the Fully Adjusted Models (Model 3), Among 3.7 Million Subjects in the PACES Cohort eMethods. Supplementary Methods [file jamanetwopen-e230561-s001.pdf]

Supplemental Online Content

Alexeeff SE, Deosaransingh K, Van Den Eeden S, Schwartz J, Liao NS, Sidney S. Association of long-term exposure to particulate air pollution with cardiovascular events in California. *JAMA Netw Open.* 2023;6(2):e230561. doi:10.1001/jamanetworkopen.2023.0561

eFigure 1. Survival Analysis Study Designs Illustrating Age as Time Scale, Time on Study, Baseline Air Pollution Exposure, and Time-Updated Air Pollution Exposure

eFigure 2. PACES Estimated CVD Mortality Effect Compared to Previous Studies in a Recently Published Meta-analysis

eFigure 3. PACES Estimated IHD Mortality Effect Compared to Previous Studies in a Recently Published Meta-analysis

eFigure 4. PACES Estimated Incident AMI Effect Compared to Previous Studies in a Recently Published Meta-analysis

eTable 1. Sensitivity Analyses Showing the Relative Risk of Each Outcome Associated With a 10 µg/m3 Increase in 1-Year Mean PM2.5 Exposure for Different Models

eTable 2. Follow-up Time in Years, Overall and by Age and SES

eTable 3. Relative Risk of Each Outcome Associated With 1-Year Mean PM2.5 Exposure, Overall and by Subgroups for Age, Sex, Race, Neighborhood Education, and Smoking Status

eTable 4. Sensitivity Analysis of the Relative Risk of Incident AMI, IHD Mortality, Cardiovascular Mortality, and Associated With 1-Year Mean PM2.5 Exposure, in

© 2023 Alexeeff SE et al. *JAMA Network Open.*

Quartiles of Neighborhood Median Income, Among 3.7 Million Subjects in the PACES Cohort

eTable 5. Relative Risk of Cardiovascular Mortality, IHD Mortality, and Incident AMI Associated With 1-Year Mean PM2.5 Exposure, in Categories Above and Below the Regulation Limit, Among 3.7 Million Subjects in the PACES Cohort

eTable 6. Assessment of Linearity of Associations by Adding a Quadratic Term for 1-Year Mean PM2.5 Exposure to the Linear Associations in the Fully Adjusted Models (Model 3), Among 3.7 Million Subjects in the PACES Cohort

eMethods. Supplementary Methods

This supplemental material has been provided by the authors to give readers additional information about their work.

© 2023 Alexeeff SE et al. *JAMA Network Open.*

eFigure 1. Survival analysis study designs illustrating age as time scale, time on study, baseline air pollution exposure, and time-updated air pollution exposure.

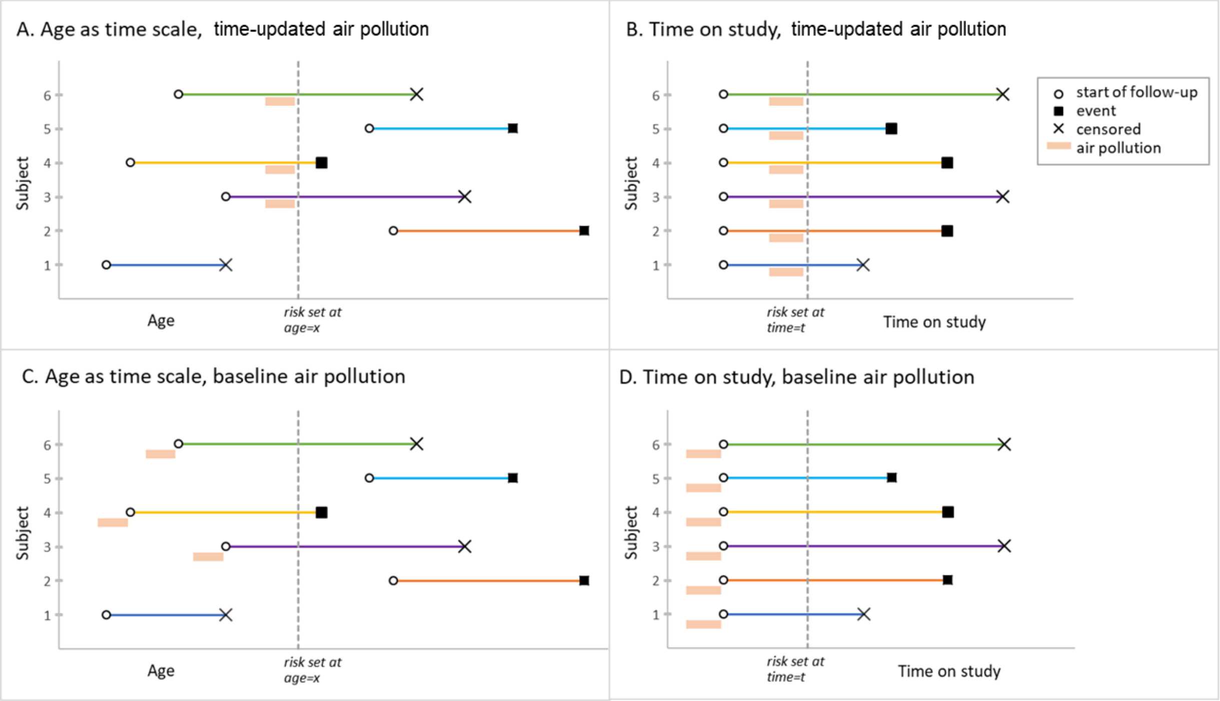

Our study used age as time scale and time-updated air pollution (A), which aligns subjects by age and appropriately computes risk estimates using the most recent 1-year mean air pollution. In this example, the risk set at age=*x* includes three subjects, and the estimated risk of the event at that instance is computed based on each subjects' mean air pollution during the preceding year. Panel (B) illustrates time on study and time-varying air pollution. In this example, the risk set at time=*t* includes all six subjects, and the estimated risk of the event at that instance is computed based on each subjects' mean air pollution during the preceding year. Panel (C) illustrates age as time scale and baseline air pollution. In this example, the risk set at age=*x* includes three subjects, and the estimated risk of the event at that instance is computed based on each subjects' mean air pollution during the year preceding the start of follow-up. Panel (D) illustrates time on study and baseline air pollution. In this example, the risk set at time=*t* includes all six subjects, and the estimated risk of the event at that instance is computed based on each subjects' mean air pollution during the year preceding the start of follow-up.

© 2023 Alexeeff SE et al. *JAMA Network Open.*

eFigure 2. PACES estimated CVD mortality effect compared to previous studies in a recently published meta-analysis.

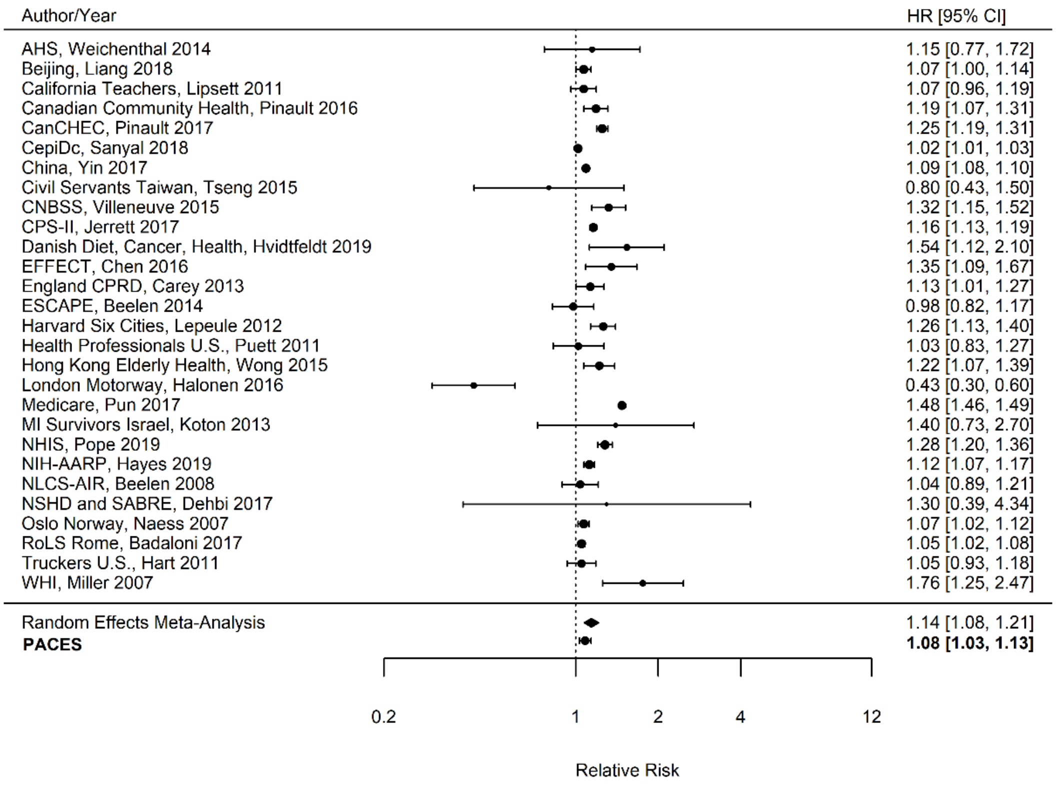

© 2023 Alexeeff SE et al. *JAMA Network Open.*

eFigure 3. PACES estimated IHD mortality effect compared to previous studies in a recently published meta-analysis.

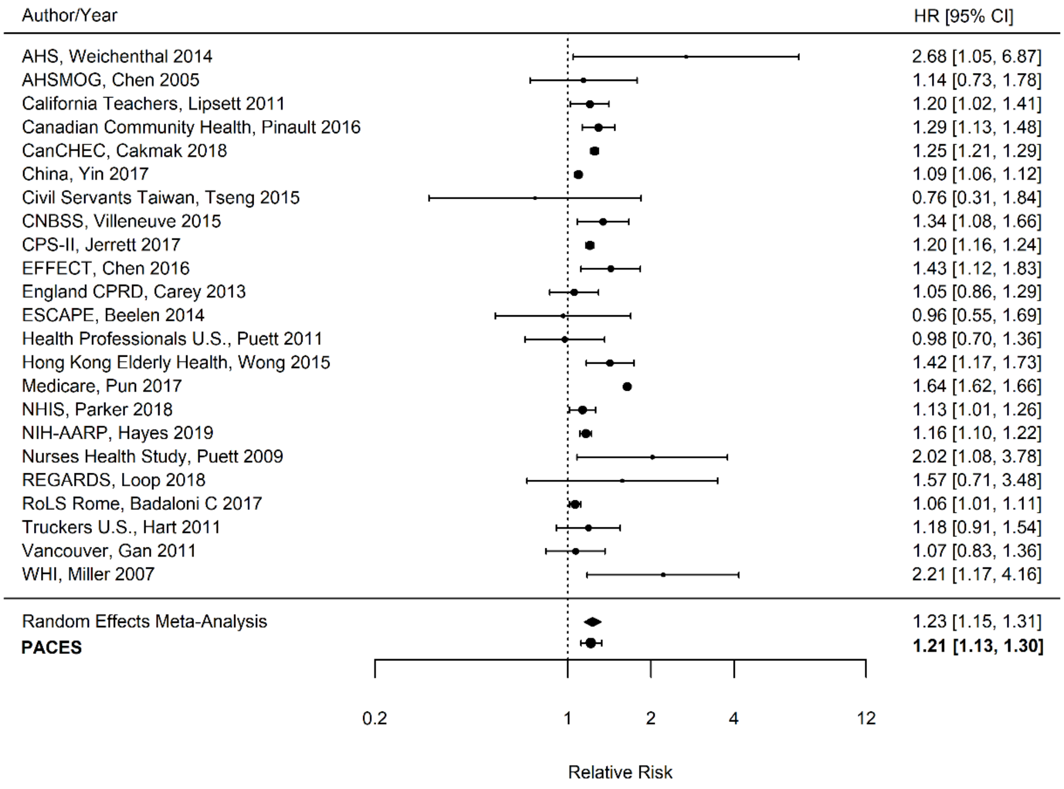

© 2023 Alexeeff SE et al. *JAMA Network Open.*

eFigure 4. PACES estimated incident AMI effect compared to previous studies in a recently published meta-analysis.

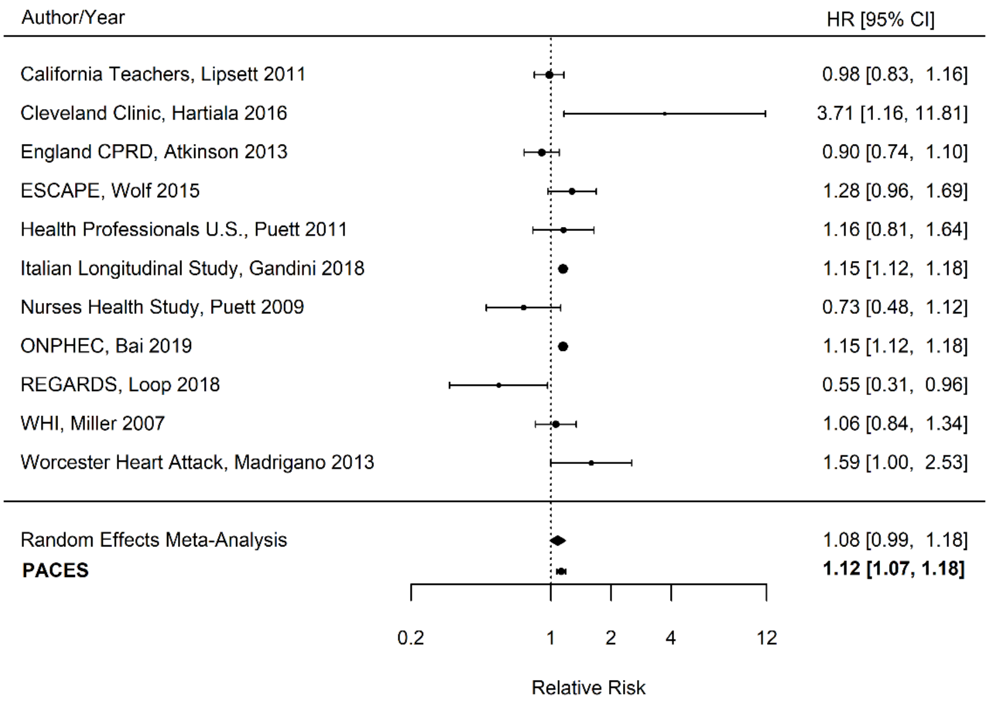

© 2023 Alexeeff SE et al. *JAMA Network Open.*

eTable 1. Sensitivity analyses showing the relative risk of each outcome associated with a 10 μg/m^3 increase in time-varying 1-year mean $PM_{2.5}$ exposure for different models.

Model	Covariates	Incident AMI (30,165 events) HR (95% CI)	IHD Mortality (14,278 events) HR (95% CI)	CVD Mortality (31,284 events) HR (95% CI)
Model 1	Age, sex, race/ethnicity	1.38 (1.32, 1.45)	1.50 (1.40, 1.60)	1.30 (1.24, 1.36)
Model 2	Age, sex, race/ethnicity, SES	1.27 (1.21, 1.33)	1.34 (1.25, 1.43)	1.18 (1.13, 1.24)
Model 2.1	Age, sex, race/ethnicity, SES, smoking	1.26 (1.20, 1.32)	1.34 (1.25, 1.44)	1.18 (1.13, 1.24)
Model 2.2	Age, sex, race/ethnicity, SES, smoking, BMI	1.24 (1.18, 1.30)	1.33 (1.24, 1.42)	1.17 (1.12, 1.23)
Model 2.3	Age, sex, race/ethnicity, SES, smoking, BMI, baseline comorbidities	1.12 (1.07, 1.18)	1.22 (1.14, 1.30)	1.08 (1.03, 1.13)
**Model 3 (fully adjusted)**	Age, sex, race/ethnicity, SES, smoking, BMI, baseline comorbidities, and baseline medication use	**1.12 (1.07, 1.18)**	**1.21 (1.13, 1.30)**	**1.08 (1.03, 1.13)**
Model 3.1	Age, sex, race/ethnicity, SES, smoking, BMI, baseline comorbidities, baseline medication use, calendar year	1.12 (1.07, 1.18)	1.21 (1.13, 1.30)	1.08 (1.03, 1.13)
Model 3.2	Age, sex, race/ethnicity, SES, smoking, BMI, baseline comorbidities, baseline medication use; uses inverse probability of censoring weighting	1.12 (1.06, 1.18)	1.23 (1.14, 1.32)	1.09 (1.03, 1.15)
Model 3.3	Age, sex, race/ethnicity, SES, smoking, BMI, baseline comorbidities, baseline medication use; adjusts for neighborhood income instead of neighborhood education	1.11 (1.06, 1.16)	1.19 (1.11, 1.27)	1.07 (1.02, 1.12)

© 2023 Alexeeff SE et al. *JAMA Network Open.*

eTable 2. Follow-up time in years, overall and by age and SES.

Subgroup	Median	25%	75%
**Overall**	4.2	1.7	9.8
**Age**			
**18-39**	2.9	1.2	6.5
**40-64**	6.2	2.2	10.0
**≥65**	8.1	3.2	10.0
**Neighborhood Education**			
**Q1 (High SES)**	4.9	1.8	10.0
**Q2**	4.6	1.8	10.0
**Q3**	4.2	1.6	9.7
**Q4 (Low SES)**	3.5	1.3	8.4

© 2023 Alexeeff SE et al. *JAMA Network Open.*

eTable 3. Relative risk of each outcome associated with 1-year mean $PM_{2.5}$ exposure, overall and by subgroups for age, sex, race, neighborhood education, and smoking status.

			Incident AMI			IHD Mortality			CVD Mortality	
**Subgroup**	**N**	**HR**	**(95% CI)**	**P Value**	**HR**	**(95% CI)**	**P Value**	**HR**	**(95% CI)**	**P-Value**
**Overall**	3,798,078	1.12	(1.07, 1.18)		1.21	(1.13, 1.30)		1.08	(1.03, 1.13)	
**Age**				0.266			0.207			0.009
**18-64**	3,118,050	1.16	(1.08, 1.25)		1.34	(1.13, 1.59)		1.25	(1.11, 1.41)	
**≥65**	680,028	1.10	(1.03, 1.17)		1.19	(1.11, 1.28)		1.05	(1.00, 1.11)	
**Sex**				0.668			0.008			0.108
**Male**	1,806,020	1.13	(1.06, 1.20)		1.12	(1.02, 1.23)		1.12	(1.05, 1.19)	
**Female**	1,992,058	1.11	(1.02, 1.20)		1.34	(1.21, 1.48)		1.04	(0.97, 1.11)	
**Race**				0.654			0.170			0.021
**White**	1,904,793	1.12	(1.06, 1.19)		1.26	(1.17, 1.37)		1.11	(1.06, 1.18)	
**Hispanic**	696,796	1.08	(0.94, 1.25)		0.98	(0.77, 1.24)		0.94	(0.80, 1.11)	
**Black**	287,980	1.09	(0.88, 1.35)		1.30	(0.97, 1.75)		1.13	(0.93, 1.38)	
**Asian/Pacific Islander**	714,043	1.24	(1.06, 1.46)		1.19	(0.89, 1.60)		1.13	(0.93, 1.38)	
**AIAN**	20,205	1.54	(0.87, 2.74)		1.28	(0.52, 3.14)		0.83	(0.41, 1.68)	
**Multiple Races**	174,261	1.07	(0.89, 1.29)		0.96	(0.74, 1.24)		0.84	(0.70, 1.00)	
**Neighborhood Education**				<0.001			<0.001			<0.001
**Q1 (High SES)**	949,562	1.03	(0.97, 1.09)		1.08	(0.99, 1.18)		0.99	(0.93, 1.05)	
**Q2**	949,434	1.10	(1.05, 1.17)		1.16	(1.08, 1.26)		1.06	(1.01, 1.20)	
**Q3**	949,569	1.16	(1.10, 1.22)		1.26	(1.17, 1.35)		1.10	(1.05, 1.16)	
**Q4 (Low SES)**	949,513	1.17	(1.10, 1.24)		1.31	(1.20, 1.43)		1.14	(1.07, 1.21)	
**Smoking**				0.051			0.199			0.396
**Never**	2,497,571	1.06	(0.99, 1.14)		1.28	(1.16, 1.41)		1.06	(0.99,1.13)	
**Current**	611,156	1.24	(1.12, 1.37)		1.26	(1.06, 1.49)		1.16	(1.03,1.32)	
**Former**	689,351	1.14	(1.05, 1.23)		1.12	(1.00, 1.25)		1.07	(0.99,1.16)	

© 2023 Alexeeff SE et al. *JAMA Network Open.*

eTable 4. Sensitivity analysis of the relative risk of incident AMI, IHD mortality, cardiovascular mortality, and associated with time-varying 1-year mean $PM_{2.5}$ exposure, in quartiles of neighborhood median income, among 3.7 million subjects in the PACES cohort.

**Outcome**	**Neighborhood Median Income**	**N**	**N events**	**HR**	**(95% CI)**	**P Value**
**Incident AMI**						0.649
	**Q1 (low SES)**	948,628	7,650	1.11	(1.05, 1.18)	
	**Q2**	950,217	7,666	1.12	(1.06, 1.18)	
	**Q3**	950,242	7,427	1.10	(1.04, 1.16)	
	**Q4 (high SES)**	948,991	7,422	1.10	(1.03, 1.18)	
**IHD Mortality**						0.001
	**Q1 (low SES)**	948,628	4,214	1.25	(1.16, 1.35)	
	**Q2**	950,217	3,735	1.21	(1.13, 1.31)	
	**Q3**	950,242	3,172	1.10	(1.02. 1.19)	
	**Q4 (high SES)**	948,991	3,157	1.11	(1.01, 1.23)	
**CVD Mortality**						0.007
	**Q1 (low SES)**	948,628	8,966	1.11	(1.05, 1.17)	
	**Q2**	950,217	7,985	1.07	(1.02, 1.13)	
	**Q3**	950,242	7,142	1.02	(0.97, 1.08)	
	**Q4 (high SES)**	948,991	7,191	1.02	(0.95, 1.09)	

eTable 5. Relative risk of cardiovascular mortality, IHD mortality, and incident AMI associated with time-varying 1-year mean $PM_{2.5}$ exposure, in categories above and below the regulation limit, among 3.7 million subjects in the PACES cohort.

**PM2.5 exposure category**	**Incident AMI HR (95% CI)**	**IHD Mortality HR (95% CI)**	**Cardiovascular Mortality HR (95% CI)**
<8.0	1.00 (ref)	1.00 (ref)	1.00 (ref)
8.0- 9.9	1.06 (1.03,1.09)	1.02 (0.98, 1.07)	0.99 (0.96, 1.02)
10.0-11.9	1.06 (1.03,1.10)	1.07 (1.02, 1.12)	0.98 (0.95, 1.02)
12.0-13.9	1.10 (1.05,1.16)	1.16 (1.08, 1.25)	1.08 (1.03, 1.14)
≥14.0	1.09 (1.02,1.15)	1.19 (1.09, 1.29)	1.11 (1.05, 1.17)

Adjusted for age, sex, race/ethnicity, SES, smoking, BMI, baseline comorbidities, baseline medication use.

eTable 6. Assessment of linearity of associations by adding a quadratic term for 1-year mean $PM_{2.5}$ exposure to the linear associations in the fully adjusted models (Model 3), among 3.7 million subjects in the PACES cohort.

**PM2.5 exposure, test for non-linearity**	**Incident AMI p-value**	**IHD Mortality p-value**	**Cardiovascular Mortality p-value**
quadratic term for 1-year mean $PM_{2.5}$ exposure	0.0011	0.3318	0.0019

Adjusted for age, sex, race/ethnicity, SES, smoking, BMI, baseline comorbidities, baseline medication use.

© 2023 Alexeeff SE et al. JAMA Network Open.

© 2023 Alexeeff SE et al. *JAMA Network Open.*

eMethods. Supplementary Methods

Imputation of missing covariates. Missing data was imputed for the covariates sex (<0.1%), race/ethnicity (8.6%), smoking (5.2%), and BMI (4.7%) using the fully conditional specification (FSC) method. The FSC method accommodates both continuous and categorical variables and does not require assuming a joint distribution for all missing variables. Instead, it uses a separate conditional distribution for each imputed variable, making the approach more flexible. The variables were imputed sequentially in the order of least to most missingness. Age, sex, neighborhood education, BMI, race/ethnicity, and smoking, were used for imputation. Continuous variables used the regression method and categorical variables used the generalized logit distribution, appropriate for variables with multiple non-ordered categories.

© 2023 Alexeeff SE et al. *JAMA Network Open.*
