# Supplementary material for: Association of Long-term Exposure to Particulate Air Pollution With Cardiovascular Events in California
Source: JAMA Netw Open. 2023 Feb 24;6(2):e230561. doi: 10.1001/jamanetworkopen.2023.0561 (PMC9958530; doi:10.1001/jamanetworkopen.2023.0561)
Supplement: Supplement 2. — Data Sharing Statement [file jamanetwopen-e230561-s002.pdf]

Data Sharing Statement

Alexeeff. Association of Long-term Exposure to Particulate Air Pollution With Cardiovascular Events in California. *JAMA Netw Open*. Published February 24, 2023. doi:10.1001/jamanetworkopen.2023.0561

Data
Data available: No

Additional Information
Explanation for why data not available: These data include protected health information. Researchers interested in collaborating on future studies analyzing this data are welcome to contact us.
